# Agronomic management enhances nutritional quality and functional food potential of purslane (*Portulaca oleracea* L.): a review

**DOI:** 10.3389/fpls.2026.1898609

**Published:** 2026-07-29

**Authors:** Peter A.Y. Ampim, Eniola A. Faluyi, Monsuru A. Salisu, Andrea McDonald, Angela Branch-Vital, Janet Antwi

**Affiliations:** 1 College of Agriculture, Food, and Natural Resources (CAFNR) Research, CAFNR, Prairie View A&M University, Prairie View, TX, United States; 2 School of Public and Allied Health, Prairie View A&M University, Prairie View, TX, United States

**Keywords:** functional foods, nitrogen management, nutritional quality, omega-3-fatty acid, purslane

## Abstract

The growing global challenges of food insecurity posed by climate change, and micronutrient deficiencies have intensified interest in climate resilient, nutrient-dense functional foods. Purslane (*Portulaca oleracea* L.) is a promising candidate owing to its exceptional nutritional profile and adaptability to harsh agroecological conditions. This review provides a comprehensive and critical synthesis of existing literature on the agronomic management, nutritional composition, and functional food potential of purslane, with particular emphasis on its omega-3 fatty acid content and the role of nitrogen and mineral nutrition. The review reveals that purslane is a rich source of alpha-linolenic acid (ALA), vitamins (C and E), carotenoids, minerals, and diverse bioactive compounds with antioxidant and anti-inflammatory properties. However, its nutritional quality is highly variable and influenced by genotype, environmental conditions, cultivation systems, and nutrient management strategies. Nitrogen plays a dual role of enhancing biomass production while exerting complex, compound-specific effects on nutrient density, often leading to trade-offs between yield and quality. The novelty of this study lies in its integrated, multi-dimensional perspective that links agronomy, plant physiology, and nutritional science, moving beyond fragmented analyses in existing literature. It highlights the importance of genotype-by-environment interactions and introduces the concept of “nutritional efficiency” as a more appropriate framework than yield-focused optimization. This study contributes to knowledge by identifying critical gaps and proposing a systems-based approach for optimizing purslane as a sustainable, plant-based source of omega-3 fatty acids and functional nutrients, particularly in resource-constrained and climate-vulnerable regions.

## 1 Introduction

The world is currently grappling with significant threats driven by human activity and a changing climate. From rising temperatures to a sharp decline in biodiversity, these challenges pose a severe risk to our health, food security, and environmental stability (Yuan et al., 2024). During the past decade, there has been great interest in the study of functional foods and their importance in addressing the challenges of food security (Valieva, 2025). Functional foods include ingredients or dishes that have specific properties that favorably affect health and reduce the risks of various kinds of diseases (Essa et al., 2023). Functional foods can directly raise population nutrient intake by delivering concentrated vitamins, minerals, and bioactive compounds targeted to deficiencies and chronic disease risk (Vlaicu et al., 2023). In this context, plant-based functional foods have gained prominence due to their accessibility, sustainability, and alignment with dietary transitions toward healthier and more environmentally conscious consumption patterns (Jiang et al., 2021; McClements and Grossmann, 2021). However, despite the recognized importance of plant-derived nutrients, significant gaps remain in the availability of certain essential compounds, particularly long-chain omega-3 fatty acids, within conventional plant-based diets (Derbyshire et al., 2024).

Conventional leafy vegetables have several limitations in delivering omega-3 fatty acids, primarily because they contain shorter-chain polyunsaturated fatty acids that are less bioavailable and harder for the body to convert into the long-chain forms found in fish. While leafy greens are a healthy source of ALA (alpha-linolenic acid), they are not a substitute for EPA and DHA (long-chain omega-3s) due to these biological constraints (Drummond, 2024; Steiner-Zitzenbacher et al., 2026). This limitation presents a critical nutritional challenge, particularly in low- and middle-income countries where access to marine-based omega-3 sources is constrained by cost, availability, or dietary preferences (Kılınç et al., 2025). Consequently, identifying alternative, plant-based sources of omega-3 fatty acids that can be integrated into local food systems remains a priority for both nutrition science and agricultural innovation.

**Figure 1:**
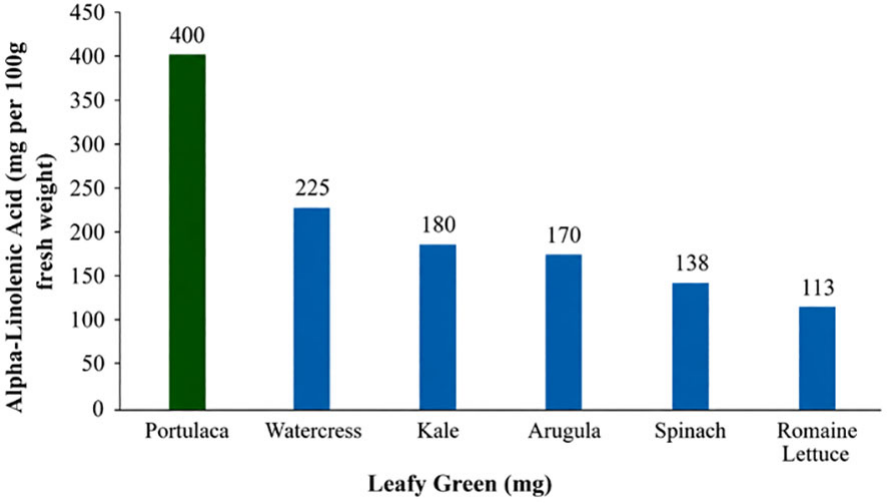
Omega-3-fatty acid (ALA) contents of popular leafy greens vs purslane. Source: (Simopoulos et al., 1992).

Purslane (*Portulaca oleracea* L.), commonly known as purslane, is a nutrient-dense plant rich in bioactive compounds such as omega-3 fatty acids, polyphenols, polysaccharides, and antioxidants (Li et al., 2024). Traditionally utilized for its medicinal properties, purslane has garnered scientific interest for its potential metabolic benefits, particularly in weight management and lipid profile improvement (Ahmed et al., 2025). The plant’s high content of alpha-linolenic acid, flavonoids, and other phytochemicals has been associated with hypolipidemic, anti-inflammatory, and anti-obesity effects (Ahmed et al., 2025). The high alpha-linolenic acid in purslane compared to other leafy vegetables is shown in **Figure 1**. Furthermore, the presence of bioactive compounds such as flavonoids, phenolics, alkaloids, and terpenoids enhances its antioxidant capacity and overall health-promoting properties (Petropoulos et al., 2019; Nemzer et al., 2020; Kumar et al., 2021).

To contextualize the nutritional advantage of purslane, a standard 100 g fresh-weight serving of purslane, containing approximately 300–400 mg ALA (Simopoulos and Salem, 1986), yields an estimated EPA equivalent of only 15–32 mg once the physiological conversion efficiency of ALA to EPA (5–8% under favorable conditions) is applied; conversion to DHA is negligible (<1%). Against the EFSA NDA Panel (EFSA Panel on Dietetic Products, Nutrition and Allergies) (2012) reference intake of 250–500 mg EPA+DHA per day, this represents approximately 3–13% of the daily requirement from a single serving.

Beyond its nutritional richness, purslane exhibits exceptional tolerance to environmental stresses, which underscores its agronomic relevance in the context of climate variability (Uddin et al., 2014; Jin et al., 2016). This adaptability is partly attributed to its unique photosynthetic flexibility. Although traditionally classified as a C_4_ plant, pursalne has been shown to exhibit inducible crassulacean acid metabolism (CAM)-like characteristics under stress conditions.

The increasing recognition of purslane as both a nutrient-dense and climate-resilient crop has stimulated research across multiple domains, including agronomy, plant physiology, and nutritional science (Drummond, 2024; Steiner-Zitzenbacher et al., 2026). Nitrogen management plays a central role in purslane’s productivity and nutritional quality (Nastou et al., 2021). Nitrogen is a critical determinant of plant growth, biomass accumulation, and metabolic activity, yet its influence on secondary metabolites and lipid biosynthesis particularly omega-3 fatty acids remains insufficiently understood (Touliabah and Almutairi, 2021). Existing studies present mixed findings, with some reporting enhanced vegetative growth under increased nitrogen supply (Touliabah and Almutairi, 2021), while others indicate potential dilution effects on nutrient density or minimal impact on fatty acid composition (Nastou et al., 2021). This inconsistency highlights the need for a comprehensive synthesis of current evidence.

Furthermore, variability in genetic background, environmental conditions, and production systems complicates the interpretation of results across studies. Differences among purslane ecotypes and cultivars may significantly influence nutrient accumulation patterns, while cultivation systems ranging from open-field production to controlled environment agriculture can modify both yield and phytochemical profiles (Carrascosa et al., 2023). These interacting factors underscore the importance of adopting an integrated perspective that considers plant genetics, environmental conditions, and nutrient management simultaneously.

Against this backdrop, this review aims to provide a comprehensive and critical synthesis of the literature on purslane (*Portulaca oleracea* L.) with particular emphasis on its nutritional composition, fatty acid profile, and the impacts of nitrogen and mineral nutrition on its functional quality. This review adopts a narrative approach and relevant literature was identified through targeted searches of Web of Science, Scopus, PubMed, and Google Scholar without year restrictions. By integrating evidence across disciplines, this review seeks to bridge existing knowledge gaps and provide a foundation for future research aimed at optimizing purslane as a viable, plant-based source of omega-3 fatty acids and other health-promoting compounds, particularly in resource-constrained environments.

## 2 Taxonomy, genetic variability, and cultivar diversity

### 2.1 Taxonomic classification and phylogenetic placement

**Figure 2:**
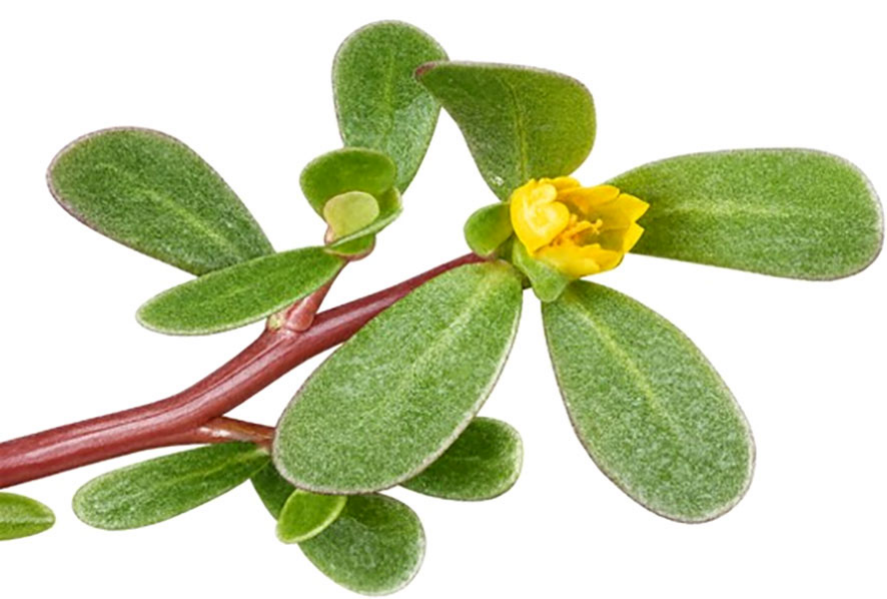
Morphological features of purslane (*Portulaca oleracea* L.). Botanical close-up of purslane showing succulent oval leaves, reddish stems, and a bright yellow flower. Source (Descouens, 2012).

Purslane (*Portulaca oleracea* L.) is a taxonomically well-defined but biologically variable species within the family *Portulacaceae*. Purslane is classified as follows: Kingdom-Plantae, Subkingdom-*Tracheobionta*, Superdivision-*Spermatophyta*, Division-*Magnoliophyta*, Class-*Magnoliopsida*, Subclass-*Caryophyllidae*, Order-*Caryophyllales*, Family- *Portulacaceae*, Genus *Portulaca*, and Species *P. oleracea* L. (OKafor et al.. 2014; Srivastava et al., 2021). This taxonomic placement is important because it situates purslane within a lineage of succulence-adapted plants that have evolved structural and physiological traits supporting survival under stressful environments. Purslane is a cosmopolitan species with smooth, prostrate, succulent stems, fleshy leaves, and small yellow flowers (**Figure 2**), features that make it readily recognizable across its diverse ecological range (Chugh et al., 2019; Hilty, 2020).

Its phylogenetic relevance goes beyond classification alone. Purslane can be described as a stress-adapted species with exceptional plasticity, especially in relation to drought, salinity, and nutrient variability (Liew et al., 2025). The species’ ability to thrive in dry and semi-dry conditions across Europe, Africa, North America, Australia, and Asia suggests that its taxonomy is best understood alongside its adaptive biology rather than as a purely morphological label (Iranshahy et al., 2017; Liew et al., 2025). This broad distribution, combined with local adaptation, helps explain why *P. oleracea* L. is often described as a highly plastic species with substantial intraspecific variation (El-Bakatoushi, 2015).

### 2.2 Genetic and phenotypic variability among purslane ecotypes

Researches indicate that purslane should be interpreted as a species with considerable genetic and phenotypic variability rather than a single fixed crop type (Sdouga et al., 2020; Carrascosa et al., 2023). Across several studies, ecotypes collected from different bioclimatic zones show differences in growth habit, mineral composition, phenolic content, antioxidant capacity, and fatty acid profile (Sdouga et al., 2020). This matters because it means the nutritional value of purslane is not determined solely by species identity; it is also shaped by the ecotype, growing environment, and harvest stage (Srivastava et al., 2021). In other words, genetic background and environmental response act together to determine final composition.

Phenotypic variability is visible in morphological traits as well as biochemical traits. Studies describe purslane as varying in stem color, leaf shape, branching habit, and growth pattern, with leaves ranging from ovate to oblong and stems ranging from light green to reddish brown depending on the variety (Chugh et al., 2019; Hilty, 2020). These are not trivial differences: in a crop valued for its leaves, small changes in leaf morphology, tissue succulence, or stem architecture can affect harvestability, consumer preference, and nutrient partitioning. More importantly, such variability is linked to differences in nutritional quality, including omega-3 fatty acids, pigments, minerals, and antioxidant compounds. For example, cultivated and wild purslane differ in fatty acids and phytochemical components (Nemzer et al., 2020), while different cultivars show distinct antioxidant properties.

Purslane also shows ecotype-level differences under environmental stress. According to Camalle et al. (2020) salinity and nitrogen sources influenced leaf quality, biomass, and metabolic responses in two ecotypes of *P. oleracea* L., reinforcing the idea that some forms are more responsive than others to management conditions. This implies that observed differences in nutritional composition may reflect not only cultivation practices but also genotype-specific responsiveness (Nemzer et al., 2020).

Hence, purslane is a genetically heterogeneous species complex in practical terms, even when formal taxonomy keeps it within a single species (Nemzer et al., 2020; Carrascosa et al., 2023). The heterogeneity is an asset for crop improvement because it creates raw material for selection, but it also complicates agronomic recommendation because a fertilizer regime that improves one ecotype may not produce the same effect in another.

### 2.3 Distinction between green and golden morphotypes (e.g., ‘goldberg golden’)

Within the genetic and phenotypic diversity of purslane, the distinction between green and golden morphotypes is especially important for both functional quality and commercial value. *Portulaca oleracea* ‘Goldberg Golden’, is an omega-3-rich annual succulent used for culinary and medicinal purposes. Grieve and Suarez (1997); Palaniswamy et al., 2000; Zhou et al. (2015), specifically identified *P. oleracea* ‘Goldberg Golden’ as a halophytic source of nutrition and medicine, which underscores the significance of this cultivar beyond a simple ornamental or market form.

Golden morphotypes like ‘Goldberg Golden’ display larger, light green to golden-yellow leaves, more upright growth (15–30 cm tall vs. prostrate green), thicker stems, and milder tart flavor, making them preferable for commercial cultivation and microgreens. Green types have higher betalain pigments and oxalate content, potentially affecting palatability, while goldens prioritize yield and visual appeal with comparable but slightly lower ALA (3-3.5 mg/g) (Carrascosa et al., 2023).

Both share superior nutrition over conventional greens, but green purslane edges in omega-3 density and vitamin E, while goldens offer better mineral bioavailability due to reduced oxalates. Green forms excel in wild resilience under salinity/drought but goldens require optimized agronomy for consistency (Uddin et al., 2014; Güven et al., 2022).

The golden morphotype is important because color often reflects underlying biochemical differences. Kopsell et al. (2016) reported that nutritionally important pigments in purslane differ between cultivars and in response to nitrogen. Kopsell et al. (2016), also showed that nitrogen fertility influences carotenoid and chlorophyll pigments in purslane cultivars. Taken together, these studies suggest that green and golden morphotypes are not merely visually distinct; they likely differ in pigment composition, nitrogen responsiveness, and possibly consumer-relevant quality traits. This is a critical point because pigment differences may also signal variation in antioxidant potential, given the relationship between carotenoids, chlorophylls, and functional quality.

Though, morphotype distinctions should not be oversimplified. Golden cultivars may be attractive because of market appeal and uniformity, but wild or green ecotypes may sometimes show stronger stress adaptation or richer phytochemical variability. Sdouga et al. (2020) documented ecotype differences in total phenolics and antioxidant activity across Tunisian bioclimatic zones (**Table 1**). The implication is that “better” morphotypes depend on the objective that a golden cultivar may be preferred for commercial appearance and controlled production, while green ecotypes may offer broader resilience or different functional profiles.

**Table 1:** Purslane morphotype comparison: green vs. golden.

Parameters	Green morphotype (wild/ecotype)	Golden morphotype (e.g., ‘goldberg golden’)	Reference(s)
Growth Habit	Prostrate (low-growing)	Upright/Erect (15–30 cm tall)	Mahr and Spangenberg (2025)
Leaf and Stem Morphology	Smaller, darker leaves	Larger, golden leaves; thicker stems	Mahr and Spangenberg (2025)
Flavor and Palatability	More tart; higher oxalates	Milder flavor; lower oxalates	Carrascosa et al. (2023)
Omega-3 Content (ALA)	Higher density	Slightly lower (3–3.5 mg/g)	Carrascosa et al. (2023)
Micronutrient Value	Higher Vitamin E	Better mineral bioavailability	Carrascosa et al. (2023)
Pigment Composition	Higher betalains	Carotenoid/Chlorophyll focused	Kopsell et al. (2016)
Stress Adaptation	Stronger (salinity/drought)	Requires optimized agronomy	Güven et al. (2022); Uddin et al. (2014)
Phytochemical Profile	High phenolic/antioxidant variability	Uniform commercial profile	Sdouga et al. (2020)
Functional Use	Wild resilience; medicinal	Culinary; microgreens; halophytic medicine	Grieve and Suarez (1997); Palaniswamy et al. (2000); Zhou et al. (2015)

### 2.4 Implications of genetic diversity for nutritional enhancement

The genetic diversity of purslane has direct implications for nutritional enhancement. Because purslane ecotypes and cultivars vary in pigments, phenolics, mineral accumulation, fatty acid composition, and antioxidant activity, genotype selection becomes a practical route for improving nutritional quality before any agronomic intervention is applied (Uddin et al., 2014). This means purslane improvement should not rely only on fertilizer optimization; it should also include cultivar choice, ecotype screening, and potentially breeding for nutrient-dense traits.

Genetic diversity could be harnessed to enhance omega-3 fatty acid content, mineral density, and antioxidant capacity, but only if nutrient management is aligned with cultivar-specific responses. For instance, nitrogen source and rate can alter omega-3 fatty acid content in purslane, and stage of harvest can further influence polyunsaturated essential fatty acid concentrations (Montoya-García et al., 2018). However, these responses are likely mediated by the cultivar or ecotype being grown. Hence, variability across growing conditions, plant parts, and germplasm sources are important (Sdouga et al., 2020). Nutritional enhancement in purslane therefore requires a genotype-by-environment perspective rather than a one-size-fits-all approach.

## 3 Agroecological requirements and production systems

### 3.1 Environmental adaptation and growth physiology

#### 3.1.1 Temperature, radiation, and water relations

Purslane (*Portulaca oleracea* L.) is a species whose agronomic value is closely tied to its ability to perform under variable and often restrictive environmental conditions. Its global distribution across Europe, Africa, North America, Australia, and Asia is linked to its ability to thrive in warm, humid spring and summer conditions, while also persisting in dry and semi-dry environments (Iranshahy et al., 2017; Liew et al., 2025). This wide ecological range is not incidental; it reflects a crop with strong physiological flexibility and a morphology adapted to survival under stress. The fleshy leaves and prostrate stems of purslane are traits that support water conservation, survival and productivity under conditions where many leafy vegetables would decline (Chugh et al., 2019; Hilty, 2020).

Temperature response is an important part of this adaptation. Purslane germination and establishment are temperature sensitive, with optimal germination occurring up to about 25 °C and thermal death occurring at 50 °C (Zauzah and Rima, 2019). This suggests that although purslane is a robust plant once established, its early developmental stages still require a manageable thermal environment. Thermal stress and humid conditions (35 °C and 90% of humidity) may induce the protective mechanisms of the species through the increased production of proline and heat shock proteins, which significantly reduce oxidative damage and preserve photosynthetic activity (Yang et al., 2012). On the other hand, the species is sensitive to low temperatures, which define its growing period within the spring to autumn period since, under low temperatures, seed germination is not possible or plants are severely damaged (Feng et al., 2015). However, according to Saffaryazdi et al. (2022), mild cold stress could be used as a cost-effective tool to increase total phenols, flavonoids and omega-3 fatty acid content through the induction of the biosynthesis of protective secondary metabolites.

Radiation also plays a major role. Purslane as a light-responsive species that performs well in sunny, open conditions and can accumulate favorable biomass and nutritional traits under adequate light supply (Chen Y. et al., 2015; He et al., 2023). This matters because light is not simply a growth driver; it also affects photosynthetic efficiency, nutrient assimilation, and the accumulation of bioactive compounds. Indoor studies with different light intensities show that productivity and nutritional quality can vary substantially with irradiance, implying that the crop’s nutritional profile is not fixed but responsive to production conditions (Chen Y. et al., 2015; He et al., 2023).

Purslane water requirements are considered low since it is a succulent and drought-tolerant plant. Although it is primarily classified as a C_4_ plant, it is able to shift to CAM-like photosynthesis when subjected to stress and return to C_4_ metabolism when stress is relieved and plants are rehydrated (Carrascosa et al., 2023). This particular mechanism allows the species to increase its water use efficiency and adapt to drought conditions through the reduction of evapotranspiration during the day and the induction of nocturnal CO_2_ uptake (Jin et al., 2015).

Water relations are where purslane’s ecological distinctiveness becomes most evident. The drought tolerance and salinity adaption of purslane are connected to its succulent habit and its suitability for low-input systems (Uddin et al., 2012; Jin et al., 2016). Its leaves and stems store water, which helps buffer short-term moisture deficits, but Saheri et al. (2020) suggest that purslane’s response to water stress is more sophisticated than simple water storage. According to this study the species can adjust its carbon metabolism in response to drought, and this flexibility is central to its adaptation.

#### 3.1.2 CAM-like carbon assimilation and drought tolerance

A particularly important theme in purslane is the induction of CAM-like carbon assimilation under drought stress. Although purslane is generally discussed as a C_4_ plant, studies show that drought can trigger crassulacean acid-like metabolism, which is later reversed after re-watering (Chandel and Singh, 2025). This is a critical physiological insight because it demonstrates that purslane is not locked into a single photosynthetic strategy. Instead, it can alter its carbon fixation pattern when conditions become restrictive, thereby reducing water loss while preserving growth potential (Ferrari et al., 2020; He et al., 2023).

This metabolic flexibility has several implications. First, it helps explain why purslane can survive and remain productive in hot, dry environments where many leafy vegetables fail (He et al., 2023). Second, it suggests that purslane should be treated as a crop with dynamic resource-use efficiency rather than a crop with static physiological behavior (Carrascosa et al., 2023). Third, it raises an important nutritional point which is a stress response that enhances survival does not always enhance edible quality in the same way. This indicates that drought and environmental stress can alter the accumulation of omega-3 fatty acids, antioxidants, and other metabolites, implying that the most resilient plants are not always the most nutritionally optimal plants (He et al., 2021). This means purslane’s CAM-like flexibility should be viewed as both an advantage and a management challenge. It gives the plant resilience in low-water systems, but it also introduces variability in product quality. For functional food production, the key issue is therefore not only whether the plant survives drought, but whether the stress regime is mild or controlled enough to preserve desirable nutrient accumulation. This is why environmental adaptation must be coupled with cultivation strategy.

#### 3.1.3 Implications for protected and low-input systems

Purslane is a crop suited to low-input agriculture. Its tolerance of drought, salinity, and warm conditions allows it to persist with limited irrigation and reduced external inputs, which is especially important in resource-constrained regions and marginal soils (Uddin et al., 2012; Jin et al., 2016). This makes purslane relevant to climate-adaptation strategies, particularly where farmers need crops that can maintain some level of productivity under stress.

At the same time, protected systems can be used to refine purslane production rather than replace field production altogether. Since purslane responds strongly to light, water, and nitrogen management, protected cultivation can help stabilize growth and improve nutritional consistency. For example, greenhouse and indoor work have shown that environmental control allows researchers to influence growth rate, nutrient accumulation, and photosynthetic performance more precisely than in open-field systems (Chen Y. et al., 2015; He et al., 2023). This is particularly relevant when the production goal is not only biomass but also functional quality.

The broader synthesis is that purslane is well suited to protected and low-input systems for different reasons. Low-input systems exploit its hardiness and resource efficiency, while protected systems exploit its responsiveness to controlled light, temperature, and nutrient supply. These two strategies are not contradictory; they are complementary. Purslane can serve both as a resilient crop in marginal environments and as a quality-target crop in controlled environments. That dual utility is one of its strongest agronomic advantages.

### 3.2 Cultivation systems

#### 3.2.1 Field-based vs. greenhouse and containerized production

Field-based cultivation is the most traditional and accessible production route for purslane, but also the one most exposed to environmental variability. In open-field systems, the species can grow successfully with minimal intervention because of its natural tolerance to drought, salinity, and poor soils (Sdouga et al., 2020). Purslane can thrive in dry areas, produces large numbers of seeds, and grows extensively in warm conditions, all of which make it well adapted to field establishment and persistence (Chandel and Singh, 2025). This field suitability helps explain why purslane is commonly encountered as both a cultivated crop and a spontaneous weed.

However, field production is inherently less predictable than protected production. Environmental fluctuations in temperature, rainfall, solar radiation, and soil moisture can all affect growth, nutrient uptake, and leaf nutritional quality. Because purslane’s nutritional composition is strongly influenced by growth stage and environmental conditions, field production may produce higher variability in both yield and quality than more controlled systems (Uddin et al., 2012; Petropoulos et al., 2019). That variability is not necessarily negative when the objective is low-cost biomass production, but it becomes problematic when the objective is standardized functional quality.

Greenhouse production offers a different balance. Greenhouse production allows more reliable interpretation of responses in growth, mineral content, and fatty acid composition. Greenhouse studies show that indoor production can improve productivity and nutritional quality under different light intensities and durations, reinforcing the suitability of protected systems for purslane improvement (Chen Y. et al., 2015; He et al., 2023).

Containerized production extends this logic further. It allows precise control over substrate, irrigation, and nutrient delivery, which is useful for studying nutrient response and for producing high-quality crops in limited spaces (Adekunle, 2017). Growing density, harvest timing, and production environment can markedly influence yield and quality. Therefore, containerized systems can be used to optimize these variables more systematically than field systems (Karkanis and Petropoulos, 2017; Petropoulos et al., 2019). Noticeably, the main value of container production is not scale but control. It is especially appropriate when the goal is to standardize nutritional composition or to study genotype–environment interactions.

#### 3.2.2 Controlled environment agriculture and urban farming applications

Purslane (*Portulaca oleracea* L.) is well suited to Controlled Environment Agriculture (CEA) due to its rapid growth, adaptability, and responsiveness to environmental regulation. CEA systems such as greenhouses, indoor farms, and hydroponics enable precise control of light, temperature, humidity, and nutrients. This is particularly important given purslane’s sensitivity to nitrogen, light, and harvest timing, allowing optimization of both yield and nutritional quality (Chen Y. et al., 2015; He et al., 2023). Hydroponic and greenhouse systems further enhance water-use efficiency, nutrient control, and planting density, supporting high yields with improved nutritional profiles (D’Imperio et al., 2020; D'Imprio et al.., 2022). This is especially relevant for purslane, where value depends not only on biomass but also on biochemical composition, making controlled systems advantageous for targeted production.

Purslane is also well aligned with urban agriculture. Its low input requirements, compact growth, and suitability for containerized and vertical systems make it ideal for space-constrained environments (Jin et al., 2016; Karkanis and Petropoulos, 2017). Its short growth cycle and tolerance to controlled conditions reinforce its applicability in urban food systems (He et al., 2023).

However, CEA does not inherently guarantee superior nutritional quality. Outcomes depend on interactions among cultivar, light, fertilization, and harvest stage; poor alignment may yield uniform but suboptimal crops (Carrascosa et al., 2023). Thus, the key advantage of CEA lies in its capacity for precise optimization rather than environmental control alone.

## 4 Nitrogen and mineral nutrition management

### 4.1 Nitrogen uptake, assimilation, and biomass allocation

Nitrogen (N) is widely recognized as a central driver of growth performance and physiological activity in *Portulaca oleracea* L., particularly through its role in chlorophyll synthesis, protein formation, and leaf expansion. As a leafy vegetable, purslane exhibits a strong positive response to nitrogen availability, where enhanced chlorophyll content and photosynthetic capacity translate into increased vegetative biomass and marketable yield (Karkanis and Petropoulos, 2017; Petropoulos et al., 2020; Muhammad et al., 2022). However, accumulating evidence indicates that this relationship is neither linear nor universally predictive of productivity outcomes.

Empirical findings reveal that nitrogen effects on yield are highly context-dependent and often secondary to agronomic factors such as harvest timing, plant density, and genotype. For example, while nitrogen fertilization has been shown to increase yield (e.g., 15.24 t ha^-^¹ under 150 kg N ha^-^¹) Kaymak (2013), higher yields (24–25 t ha^-^¹) have also been reported under non-fertilized conditions when longer growth periods and optimized plant densities were employed (Karkanis and Petropoulos, 2017; Petropoulos et al., 2019). Similarly, Mortley et al. (2012) demonstrated exponential biomass accumulation with extended growth duration, indicating that temporal dynamics may exert a stronger influence on yield than nitrogen supply alone.

Beyond total biomass, nitrogen availability also influences biomass allocation patterns. Under elevated nitrogen conditions, purslane tends to preferentially allocate resources to shoot growth and leaf expansion at the expense of root development (Chrysargyris et al., 2023; Liang et al., 2026). While this shift enhances short-term productivity, it may reduce resilience under suboptimal conditions, particularly in water- or nutrient-limited environments. Thus, nitrogen-induced allocation patterns may reflect a trade-off between rapid above-ground growth and long-term physiological stability.

An additional layer of complexity arises from the form of nitrogen supplied. Evidence indicates that the balance between nitrate (NO_3_^-^) and ammonium (NH_4_^+^) plays a critical role in regulating growth and quality. While nitrate-dominant nutrition tends to favor higher yields, mixed or ammonium-rich regimes may enhance certain quality traits but can also induce physiological stress at higher concentrations (Carrascosa et al., 2023). This highlights that nitrogen management extends beyond quantity to include ionic composition and plant physiological responses.

These dynamics collectively underscore the importance of nitrogen use efficiency (NUE) as a multidimensional concept. In purslane, NUE should not be assessed solely in terms of biomass accumulation per unit nitrogen, but rather as a balance between growth, resource allocation, and nutritional outcomes. Although optimized nitrogen supply can enhance productivity, higher nitrogen inputs do not consistently translate into improved nutritional quality. While some studies reported reductions in certain compounds under high nitrogen (He et al., 2023), others demonstrate improvements in specific nutritional attributes, such as increased omega-3 fatty acids and reduced oxalic acid content (Montoya-García et al., 2018).

**Figure 3:**
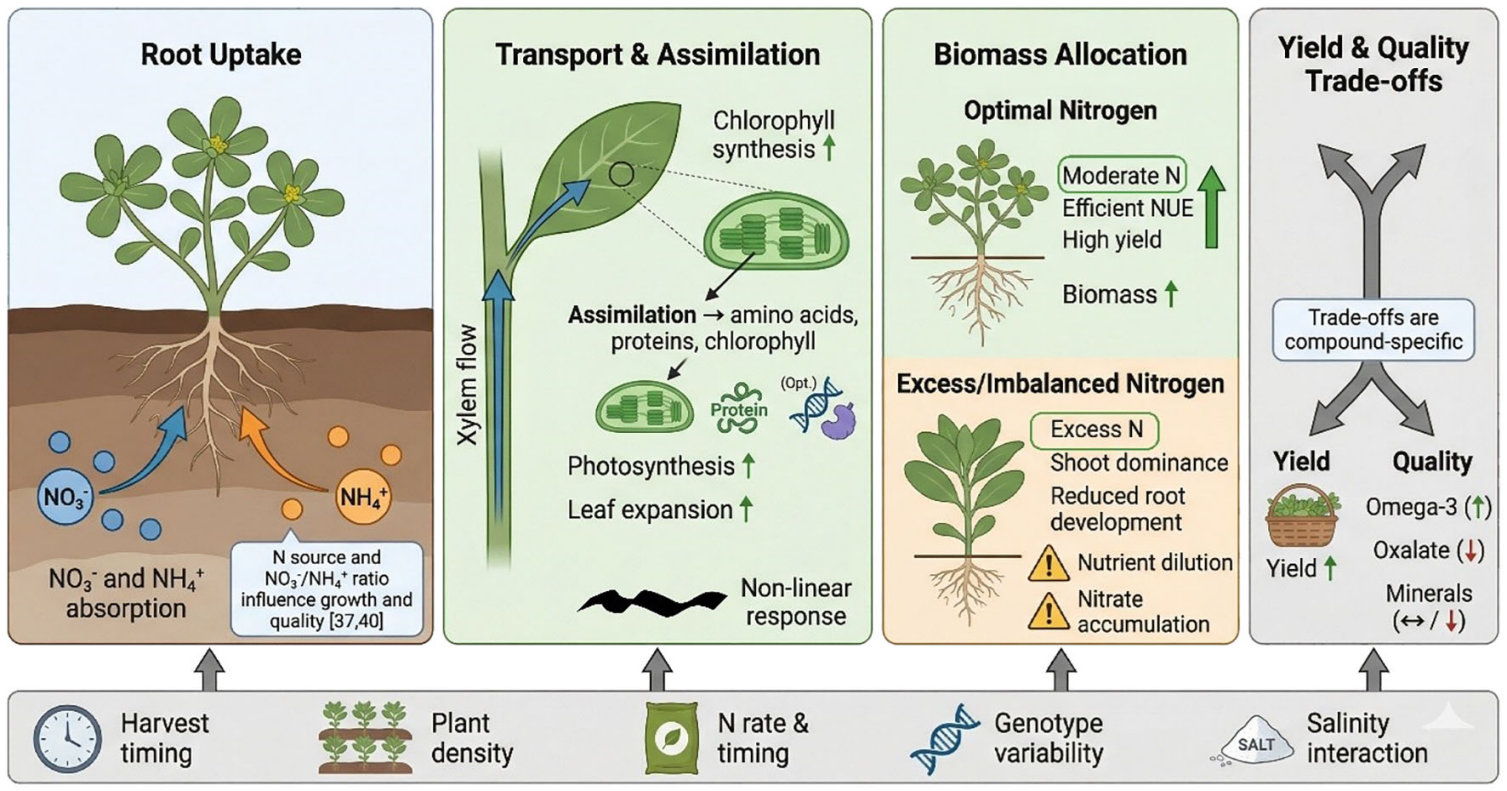
Nitrogen uptake, assimilation, and biomass allocation in purslane (*Portulaca oleracea* L). The graphical illustration shows nitrogen uptake, assimilation, and biomass allocation in purslane (*Portulaca oleracea* L). It shows the effects of nutrient levels and environmental modifiers on plant yield and quality. Source: Authors’ Creation.

Therefore, nitrogen effects on quality are compound-specific and not uniformly beneficial or detrimental, reflecting complex metabolic adjustments within the plant **Figure 3**. This reinforces the need to reconceptualize NUE in purslane as “nutritional efficiency”, where the objective is not only to maximize yield but also to optimize the concentration and composition of health-promoting compounds.

### 4.2 Mineral nutrition and nutrient interactions

Beyond nitrogen, purslane (*Portulaca oleracea* L.) demonstrates strong mineral accumulation, supporting its classification as a nutrient-dense leafy vegetable. It contains substantial macronutrients (K, Ca, Mg) and micronutrients (Fe, Zn), though their levels are highly influenced by the environment, nutrient supply, and genotype (Uddin et al., 2014; Petropoulos et al., 2020; Carrascosa et al., 2023). Mineral dynamics reflect a balance between total uptake and tissue concentration. While nitrogen can enhance biomass and overall nutrient uptake, it may reduce mineral concentration through dilution effects (Karkanis and Petropoulos, 2017; Petropoulos et al., 2019). However, it is worth noting that recent evidence shows mineral-specific and context-dependent patterns rather than consistent declines (Carrascosa et al., 2023) indicating that this response is not uniform.

Nitrogen interactions with other nutrients further shape this variability. Increased growth raises demand for K and Mg, essential for physiological functions, yet excessive nitrogen can disrupt nutrient balance and reduce micronutrient uptake (Veres et al., 2026). This highlights the interdependent nature of plant nutrition, where imbalance in one element affects overall nutrient efficiency.

Distinctions also emerge between yield and quality related nutrients. Phosphorus and potassium may have limited effects on biomass but significantly enhance bioactive compounds such as flavonoids and carotenoids (Fontana et al., 2006; Montoya-García et al., 2018). Meanwhile, higher nitrogen increases nitrate accumulation in edible tissues, though typically within safe limits and manageable through fertilization strategies (El-Sherbeny et al., 2015).

Nutrient interactions can be synergistic or antagonistic. Adequate potassium improves water-use efficiency and complements nitrogen-driven growth, whereas deficiencies in calcium or magnesium constrain plant function. Conversely, excessive nitrogen without balanced supply reduces micronutrient density (Kaspari and Welti, 2024). Environmental factors add further complexity as genotype influences nitrate accumulation, and moderate salinity enhances nutritional quality under specific nitrogen regimes (Camalle et al., 2020).

Controlled environment systems provide a pathway to manage these interactions precisely, enabling optimized nutrient solutions and improved crop quality (D’Imperio et al., 2020; He et al., 2021; D'Imprio et al., 2022). Thus, while purslane is inherently adaptable, maximizing its nutritional potential requires integrated and balanced nutrient management.

## 5 Nutritional composition and nutrient density

### 5.1 Proximate composition

#### 5.1.1 Protein, dietary fiber, carbohydrates, and ash content

The proximate composition of purslane (*Portulaca oleracea* L.) positions it as a nutritionally exceptional leafy vegetable, particularly when benchmarked against commonly consumed greens such as spinach and lettuce. However, a critical appraisal of the available literature reveals that the nutritional values reported across studies are highly variable, reflecting the compounding influence of genotype, plant part, growth stage, and environmental conditions a complexity that demands caution when making generalized nutritional claims.

In terms of protein content, studies reveals that the plant’s nutritional density is heavily influenced by the analytical state (fresh versus dry weight), the morphological part of the plant, the harvesting stage, and the environmental conditions. When evaluating the protein content of purslane, it is critical to account for whether the analysis was conducted on a fresh weight (FW) or dry weight (DW) basis, as purslane is highly succulent (Gabr et al., 2021). For example, He et al. (2021) equally reported that Egyptian *P. oleracea* L. leaves contain 12.13% crude protein on a dry weight basis but a study by El Gindy (2017) highlights immense variability in this metric, noting a protein level of 15.27%, and as high as 23.47% in the same Egyptian cultivar. This wide range suggests that genetic variety, vigor, and the environmental conditions during maturation heavily dictate protein accumulation. Studies analyzing fresh plant material report lower nominal values. Fukalova et al. (2022) observed 1.49% FW in cultivated purslane and 1.56% FW in wild purslane, indicating no statistically significant difference in protein yield based on these growing environments.

The nitrogen-dependent nature of protein synthesis means that nitrogen fertilization directly modulates these values. Studies have demonstrated that elevated nitrogen supply, particularly from ammonium sources, significantly increases protein accumulation in purslane leaves, while excessive nitrogen beyond an optimal threshold reduces water-soluble proteins in shoots suggesting a non-linear relationship between nitrogen availability and protein yield (Szalai et al., 2010; de Jesus et al., 2016). This has direct agronomic implications in that nitrogen management cannot be optimized for biomass alone without regard for its downstream effects on protein quality and amino acid composition.

Dietary fiber content in purslane, while modest on a fresh weight basis (1.8 to 4.1 g per 100 g FW), is predominantly insoluble in character and thus physiologically relevant to bowel function, cholesterol reduction, and glycemic modulation (Dhingra et al., 2012; Ramos et al., 2026; USDA, 2023). Notably, purslane contains pectin and resistant starch soluble fiber fractions that confer additional prebiotic and postprandial glucose-regulatory benefits (Petropoulos et al., 2020). Some studies report substantially higher fiber values when expressed on a dry weight basis (up to 21.6 g/100 g DW), though these figures are not directly comparable to fresh weight data without moisture correction (Fukalova et al., 2022). However, it is highly notable that cultivated *P. oleracea* L. exhibits extreme instability in its fiber content, displaying a coefficient of variability of 34.23%, compared to a mere 0.42% variance in wild species (Fukalova et al., 2022). This suggests that while organic farming can slightly boost the nominal fiber average, standardized agricultural practices do not yield highly uniform fiber accumulation in this plant (Fukalova et al., 2022). To place these proximate values into a nutritional context, Fukalova et al. (2022) critically evaluated *P. oleracea* L. against conventional fresh greens. While purslane’s fresh fiber content (approx. 2.4-2.6%) does not match highly fibrous greens like parsley (7.26 g/100g FW), it remains an essential functional food component recognized for improving digestion and mitigating the risks of diabetes, atherosclerosis, obesity, and constipation.

The carbohydrate profile of purslane is characterized by a low total carbohydrate content of approximately 3–4% of fresh weight, rendering it suitable for low-carbohydrate dietary patterns, including ketogenic regimens (Simopoulos, 2013; USDA, 2024). The principal carbohydrate fractions are simple sugars glucose, fructose, and sucrose which contribute to the plant’s mildly sweet and tangy palatability (Ilieva et al., 2015). A notable ontogenetic shift in carbohydrate distribution occurs during plant development. At 29 days after sowing (DAS), stems contain higher carbohydrate concentrations (7.6 g/100 g FW) compared to leaves (5.13 g/100 g FW). This gradient reverses by 52 DAS because stems exhibit declining concentrations and while leaves increase to 6.2 g/100 g FW (Petropoulos et al., 2019). This temporal and tissue-specific variation has practical consequences for producers, suggesting that harvest timing can be strategically selected to target specific carbohydrate fractions within the plant.

Ash content a proxy for total mineral load ranges from 1.3 to 2.2 g per 100 g FW in most studies, with some dry weight reports reaching 22.66% (Aberoumand, 2009; USDA, 2023). Fukalova et al. (2022) reported an exceptionally high ash value of 17.8 g/100 g FW. This observation may reflect methodological differences in moisture correction or plant material selection. The relatively high ash content of purslane, compared to many conventional leafy vegetables, reflects its capacity for mineral accumulation which is a function of both its succulent physiology and the mineral richness of its growth substrate **Table 2**. Given that ash content is directly influenced by nitrogen fertilization regime, soil mineral availability, and harvest stage, standardized reporting protocols are critically needed to enable meaningful cross-study comparisons (Carrascosa et al., 2023).

**Table 2:** Summary of the proximate composition of *Portulaca oleracea* L. across different studies.

Reference	Plant Part and condition	Measurement basis	Protein (%)	Crude Fiber (%)	Carbohydrates (%)	Ash (%)
Gabr et al. (2021)	Leaves (Grown in Egypt)	Dry Weight	12.13	7.24	55.41	19.39
Fukalova et al. (2022)	Leaves and Tender Stems (Wild)	Fresh Weight	1.56	2.39	4.72	2.62
Fukalova et al. (2022)	Leaves and Tender Stems (Cultivated)	Fresh Weight	1.49	2.60	8.41	3.39
Petropoulos et al. (2019)	Stems (29 Days After Sowing)	Fresh Weight	1.31	*Not reported*	7.60	2.48
Petropoulos et al. (2019)	Leaves (29 Days After Sowing)	Fresh Weight	1.57	*Not reported*	5.13	2.14
Petropoulos et al. (2019)	Stems (43 Days After Sowing)	Fresh Weight	0.76	*Not reported*	5.40	1.78
Petropoulos et al. (2019)	Leaves (43 Days After Sowing)	Fresh Weight	1.91	*Not reported*	5.25	1.89
Petropoulos et al. (2019)	Stems (52 Days After Sowing)	Fresh Weight	1.44	*Not reported*	5.39	1.74
Petropoulos et al. (2019)	Leaves (52 Days After Sowing)	Fresh Weight	2.96	*Not reported*	6.20	2.40

Values are reported on either a fresh-weight (FW) or dry-weight (DW) basis.

#### 5.1.2 Variability due to genotype and environment

A fundamental limitation in the nutritional characterization of purslane is the profound inter-study variability attributable to genotype-by-environment interactions. The nutritional composition including protein, fiber, ash, moisture, and carbohydrate content differs substantially between wild and cultivated ecotypes, between leaves and stems, and across harvest stages (Petropoulos et al., 2019). Wild specimens consistently report higher moisture content and distinct phytochemical profiles compared to their cultivated counterparts. Cultivated varieties often benefit from agronomic inputs that can be tailored to optimize specific nutritional components (Fukalova et al., 2022). Ornamental varieties, such as the ‘Goldberg Golden’ morphotype, have been shown to contain lower total mineral content compared to common purslane, highlighting that variety selection is not merely an aesthetic choice but one with measurable nutritional consequences (Carrascosa et al., 2023).

Environmental variables including light intensity, temperature regime, soil composition, water availability, and nitrogen supply collectively govern the partitioning of carbon and nitrogen within the plant, thereby modulating proximate composition. For instance, plants cultivated under high light conditions accumulate more carbohydrates through enhanced photosynthesis, while those subjected to saline stress may exhibit elevated protein content as part of an osmotic stress response (Giménez et al., 2021). The absence of standardized production protocols across published studies makes it challenging to isolate the contribution of any single agronomic factor. Hence future nutritional assessments of purslane could benefit substantially from replicated trials under controlled conditions with clearly defined cultivar identities and growth parameters.

### 5.2 Mineral and vitamin profile

#### 5.2.1 Bioavailable forms of Ca, Mg, Fe, Zn, and P

The mineral profile of purslane (*Portulaca oleracea* L.) represents one of its most compelling nutritional attributes. A critical assessment of its profile must account not only for elemental abundance but also for the bioavailability of individual minerals a dimension frequently overlooked in agronomic studies of the species. Purslane is reported to contain K as its dominant mineral (up to 494 mg per 100 g FW), followed by Ca, Mg, Na, and P, with Fe, Zn, and Mn present as important trace elements (Nemzer et al., 2020; Gabr et al., 2021.; Fukalova et al., 2022; Makangali et al., 2025). However, the physiological relevance of these concentrations is contingent upon the forms in which these minerals occur and the presence of anti-nutritional factors principally oxalic acid that can complex divalent cations and reduce their intestinal absorption (Srivastava et al., 2019).

Calcium content in purslane leaves, reported at approximately 65 mg per 100 g FW (Uddin et al., 2014), is nutritionally meaningful for bone structural integrity, cell signalling, and neuromuscular function (Popescu et al., 2018). Nitrogen fertilization has been demonstrated to significantly influence Ca accumulation in purslane leaves. Application at 15,000 mg N/m^2^ produced the highest Ca concentrations compared to unfertilized controls in greenhouse studies, suggesting that judicious nitrogen management can enhance this mineral fraction (Chrysargyris et al., 2024). However, the co-occurrence of oxalic acid present at 671–869 mg per 100 g FW in purslane leaves (Uddin et al., 2014) raises an important question on Ca bioavailability: whether it will be substantially diminished through the formation of insoluble calcium oxalate (CaC_2_O_4_) complexes. Processing interventions such as boiling or steaming have been shown to reduce oxalic acid content, thereby potentially improving Ca bio-accessibility for consumers who rely on purslane as a dietary calcium source (Srivastava et al., 2019).

Magnesium is present at approximately 68 mg per 100 g FW (Uddin et al., 2014). Nitrogen management has a significant impact on magnesium accumulation in leafy vegetables, with responses controlled by biomass-driven dilution effects, nitrogen availability, and nutrient interactions. A balanced nitrogen nutrition promotes better nutritional quality in leafy vegetables, while excessive nitrogen fertilization may lower mineral concentrations in edible tissues due to accelerated vegetative growth and changed cation uptake dynamics (Kyriacou et al., 2016; Rouphael et al., 2018).

Iron content in purslane approximately 1.99 mg per 100 g FW (Uddin et al., 2014) is notable relative to other leafy greens and is physiologically critical for hemoglobin synthesis, oxygen transport, and enzymatic activity. Despite its functional importance, Fe concentration in purslane leaves was not significantly affected by nitrogen fertilization rates in greenhouse experiments (Santos et al., 2016), suggesting that Fe homeostasis in this species may be tightly buffered against changes in nitrogen supply.

Zinc, present at concentrations that support immune function, wound healing, protein synthesis, and DNA integrity (Okafor et al., 2014), exhibited a notably declining trend with increasing nitrogen application in greenhouse studies with Zn content decreasing significantly as nitrogen rate increased from 0 to 22,500 mg N/m^2^ (Montoya-García et al., 2018). This inverse relationship between nitrogen rate and Zn concentration is consistent with a nutrient dilution effect mediated by accelerated biomass accumulation, wherein the rate of dry matter accumulation outpaces Zn uptake and redistribution (Chrysargyris et al., 2024). For Zn as for Mn which similarly declined at the highest nitrogen rate, this represents a genuine nutritional trade-off. This is because the same nitrogen regimes that maximize vegetative yield may simultaneously reduce the concentration of these micronutrients per unit biomass. This has practical implications for improving the quality of functional foods because these challenges are rarely addressed in agronomic optimization studies.

Phosphorus, as a constituent of ATP, nucleic acids, and phospholipid cell membranes, is essential for energy metabolism and plant and human cellular integrity (Stigter and Plaxton, 2015). Its accumulation in purslane leaves was significantly enhanced at 15,000 mg N/m^2^ compared to the unfertilized control and 7,500 mg N/m^2^ treatments (Chrysargyris et al., 2024), a synergistic nutrient interaction that may reflect nitrogen-stimulated root activity and increased P uptake capacity. The form of nitrogen fertilizer further modulates P dynamics: application of calcium nitrate has been associated with slightly higher P concentrations in purslane (2.9 mg P/g) compared to ammonium-based sources (2.4–2.5 mg P/g), indicating that nitrogen source selection has nutritional consequences beyond nitrogen alone (Makangali et al., 2025). **Table 3** contains mineral compositions of purslane across different studies under different conditions: Dry Weight (DW) vs. Fresh Weight (FW). Minerals measured on a fresh weight basis (Fukalova et al., 2022) appear significantly lower in absolute mass because fresh purslane contains 83% to 92% water. The study by Uddin et al. (2014) measures minerals in millimoles per kilogram (mmol/kg) rather than milligrams, tracking how these concentrations change as the plant matures from 15 days to 60 days after sowing.

**Table 3:** Mineral composition of **Portulaca oleracea*.* L across multiple studies.

Mineral	Gabr et al. (2021) (mg/100g DW)	Nemzer et al. (2020) (mg/100g DW) cultivated/wild	Makangali et al. (2025) (mg/100g DW)	Fukalova et al. (2022) (mg/100g FW) cultivated/wild	Uddin et al. (2014) (mmol/kg DW) range (15 to 60 days)
Potassium (K)	1283.50	6400.00/6690.00	4643.4	271.91/776.67	1257 increases to 1526
Calcium (Ca)	793.35	914.33/1033.33	765.6	110.59/186.67	1612 increases to 1945
Magnesium (Mg)	517.56	1266.67/916.33	1595.4	91.68/165.33	2127 increases to 2443
Sodium (Na)	17.06	35.07/15.57	298.8	0.81/16.60	356 *decreases to* 278
Phosphorus (P)	*Not reported*	281.67/580.33	376.0	58.73/33.67	*Not reported*
Iron (Fe)	10.39	41.73/121.67	531.0	1.35/1.80	218 increases to 262
Zinc (Zn)	14.17	6.62/5.26	6.5	1.08/0.99	128 increases to 160
Manganese (Mn)	0.57	6.77/6.08	12.6	*Not reported*	*Not reported*
Copper (Cu)	*Not reported*	1.18/1.20	*Not reported*	0.36/0.14	*Not reported*
Selenium (Se)	*Not reported*	0.0066/0.0101	0.09	*Not reported*	*Not reported*
Chloride (Cl)	*Not reported*	*Not reported*	*Not reported*	*Not reported*	82 *decreases to* 53

Values are reported on either a fresh-weight (FW) or dry-weight (DW) basis.

#### 5.2.2 Vitamin C, carotenoids, and tocopherols

Purslane is recognized as a rich source of several bioactive vitamins notably ascorbic acid (vitamin C), alpha-tocopherol (vitamin E), and beta-carotene (provitamin A) whose combined antioxidant action confers significant health-protective properties (Uddin et al., 2012; Burri et al., 2017). Vitamin C (ascorbic acid) in purslane, reported at approximately 21 mg per 100 g FW (Uddin et al., 2014), contributes meaningfully to its antioxidant capacity and enhances non-heme iron bioavailability. Its role in overall antioxidant activity is supported by assays such as DPPH, hydrogen peroxide scavenging, and FRAP (Alam et al., 2014a). However, ascorbic acid is highly sensitive to heat, oxidation, and prolonged storage, making post-harvest handling a critical determinant of its retention. Processing methods therefore play a key role, with freeze-drying shown to preserve antioxidant properties more effectively than conventional hot-air drying, which significantly reduces ascorbic acid and related phytochemicals (Youssef and Mokhtar, 2014).

Purslane also contains a diverse carotenoid profile, dominated by beta-carotene (≈3.5 mg per 100 g FW), along with lutein and zeaxanthin at nutritionally relevant levels (Li et al., 2024). These compounds contribute both to provitamin A activity and antioxidant defence as well as its established roles in protecting against oxidative stress and supporting eye health (Black et al., 2020). Importantly, carotenoid accumulation appears responsive to agronomic practices, particularly nitrogen supply, with evidence indicating increased concentrations of beta-carotene and lutein under higher nitrogen regimes (Kopsell et al., 2016). This suggests a potential alignment between yield optimization and enhancement of specific antioxidant pigments.

Alpha-tocopherol (vitamin E), present at appreciable levels in purslane (Uddin et al., 2014), further strengthens its antioxidant profile by protecting polyunsaturated fatty acids such as alpha-linolenic acid from oxidative degradation (Petropoulos et al., 2019). Its concentration tends to increase at later harvest stages, indicating that plant maturity influences tocopherol accumulation. However, this pattern introduces a trade-off, as earlier harvests are often associated with higher omega-3 fatty acid content. Consequently, optimizing harvest timing requires balancing different nutritional attributes, highlighting the broader complexity of maximizing functional quality in purslane. **Table 4** shows the vitamin C, carotenoids, and tocopherols of purslane across multiple studies and under different conditions.

**Table 4:** Vitamin C, carotenoids, and tocopherols contents of **Portulaca oleracea*.* L.

Compound	Source/citation	Plant part and condition	Reported content (with units)
Vitamin C (Ascorbic Acid)	Nemzer et al. (2020)	Whole Plant (Cultivated)	152 mg/100 g (Dry Weight)
	Nemzer et al. (2020)	Whole Plant (Wild)	140 mg/100 g (Dry Weight)
	Uddin et al. (2014)	Shoots (15 to 60 days of growth)	86.5 down to 60.5 mg/100 g (Dry Weight)
	Makangali et al. (2025)	Whole Plant (Grown in Arid Region)	20.06 mg/100 g
	Gabr et al. (2021)	Leaves (Grown in Egypt)	0.30356 mg/100 g. (Dry Weight)
Carotenoids	Aoudeh et al. (2024)	Acetone Extract	14.16 mg/g (beta-carotene, Dry Weight)
	Aoudeh et al. (2024)	Methanol Extract	3.58 mg/g (beta-carotene, Dry Weight)
	Makangali et al. (2025)	Methanol Extract	4.3 mg/g (Total Carotenoids, Dry Extract)
	Nemzer et al. (2020)	Leaves (Cultivated)	2.47 mg/g (Total Carotenoids as Lutein)
	Nemzer et al. (2020)	Leaves (Wild)	2.37 mg/g (Total Carotenoids as Lutein)
Tocopherols (Vitamin E)	Nemzer et al. (2020)	Whole Plant (Cultivated)	11.97 mg/100 g (alpha-tocopherol, Dry Weight)
	Nemzer et al. (2020).	Whole Plant (Wild)	7.793 mg/100 g (alpha-tocopherol, Dry Weight)
	Petropoulos et al. (2019)	Leaves (29 to 52 days of growth)	0.302–0.481 mg/100 g (Total Tocopherols, Fresh Weight)
	Petropoulos et al. (2019)	Stems (29 to 52 days of growth)	0.0174–0.0447 mg/100 g (Total Tocopherols, Fresh Weight)
	Petropoulos et al. (2019)	Leaves (Across harvest time points)	0.197–0.327 mg/100 g (alpha tocopherol, Fresh Weight)
	Gabr et al. (2021)	Leaves (Grown in Egypt)	0.27415 mg/100 g (Dry Weight)
	Makangali et al. (2025)	Whole Plant (Grown in Arid Region)	< 0.1 mg/100 g (Below detection limit)
	Uddin et al., 2014	Leaves	22.2 mg/100 g (Fresh Weight)

Values are reported on either a fresh-weight (FW) or dry-weight (DW) basis.

## 6 Fatty acid biosynthesis and lipid profile

### 6.1 Fatty acid composition of purslane tissues

The lipid profile of *Portulaca oleracea* L. is its most nutritionally distinctive feature, setting it apart from most leafy vegetables. Although total lipid content is low about 0.36 g/100 g fresh weight and 6.90 g/100 g dry weight (Petropoulos et al., 2019) its quality is exceptional. The lipid fraction is dominated by polyunsaturated fatty acids (PUFAs), particularly omega-3s, highlighting the need to clearly distinguish between lipid quantity and nutritional quality (Gabr et al., 2021). Fatty acid composition is highly variable, shaped by tissue type, growth stage, and environment as shown in **Table 5**. Leaves consistently contain higher PUFA levels, with α-linolenic acid (ALA) comprising 35.4–54.92% of total fatty acids, whereas stems show lower ALA but higher palmitic and linoleic acids (Petropoulos et al., 2019). This divergence reflects functional differences: leaves prioritize membrane-rich ALA for photosynthesis, while stems favor structural and storage lipids.

**Table 5:** Fatty acid and lipid composition of *Portulaca oleracea* L. across different studies and different plant parts.

Fatty acid/lipid category	Gabr et al. (2021) (leaves) Unit: % of total	Makangali et al. (2025) (extract) unit: % of total	Petropoulos et al. (2019) (range across 29–52 days) unit: % of total	Nemzer et al. (2020) (cultivated/wild) unit: mg/100g DW
Total Lipid Content	*Not reported*	3.5% (Total Fat)	6.90 g/100g (Dry Weight, Mediterranean accessions)	*Not reported*
Alpha Linolenic acid (ALA) *(C18:3, Omega-3 PUFA)*	47.92	26.7	Leaves: 35.40 – 54.92, Stems: 11.64 – 17.31	Leaves: 189.16/188.48, Stems: 18.09/29.86
Linoleic acid (C18:2, Omega-6 PUFA)	18.65	10.6	Leaves: 11.40 – 14.81, Stems: 23.02 – 27.11	Leaves: 51.19/56.09, Stems: 21.92/34.67
Palmitic acid (C16:0, SFA)	14.24	21.7	Leaves: 9.80 – 12.39, Stems: 20.20 – 21.80	Leaves: 54.48/52.34, Stems: 13.45/19.61
Stearic acid (C18:0, SFA)	2.70	4.5	Leaves: 2.52 – 3.89, Stems: 4.55 – 4.90	Leaves: 9.63/8.10, Stems: 2.68/5.23
Total SFA	19.41	*Not reported*	Leaves: 27.58 – 42.20, Stems: 44.82 – 54.80	Leaves: 64.11/60.44Stems: 16.13/24.84
Total PUFA	66.57	*Not reported*	Leaves: 50.50 – 66.53Stems: 37.81 – 42.94	Leaves: 240.35/244.57Stems: 40.01/64.53

Values are reported on either a fresh-weight (FW) or dry-weight (DW).

Overall, purslane lipids are dominated by four fatty acids: palmitic and stearic (saturated), and linoleic and ALA (unsaturated). In leaves, PUFA content (50.5–66.53%) exceeds saturated fatty acids (27.58–42.2%), yielding a favorable PUFA: SFA ratio aligned with dietary recommendations. In contrast, stems contain higher saturated fats (44.82–54.8%) and lower PUFA levels (37.81–42.94%) (Petropoulos et al., 2019). These differences have practical implications, suggesting that leaves are more suitable for nutritional applications, though this is rarely emphasized in dietary guidance. Beyond ALA, purslane contains trace amounts of docosahexaenoic acid (DHA), an uncommon feature in terrestrial plants (Li et al., 2024). While limited, this raises potential for developing plant-based omega-3 sources for populations with low EPA/DHA intake (Uddin et al., 2014; Petropoulos et al., 2019). Additionally, purslane seeds are rich in ALA, linoleic, and oleic acids, supporting their use in food, pharmaceutical, and cosmetic applications (Mohamed et al., 2013).

### 6.2 Alpha-linolenic acid biosynthesis and accumulation

Alpha-linolenic acid (ALA, C18:3n-3) is an essential omega-3 fatty acid that must be obtained from the diet (Ji et al., 2015; Zanetti et al., 2015). In plants, it is synthesized through sequential desaturation from palmitic acid via oleic and linoleic acids, catalyzed by omega-3 desaturases (FAD3, FAD7, FAD8) (Wasternack and Feussner, 2018). In *Portulaca oleracea* L. this pathway is highly active in leaf tissues, where ALA dominates chloroplast membrane lipids, particularly monogalactosyldiacylglycerol (MGDG) (Petropoulos et al., 2019).

Purslane exhibits exceptionally high ALA levels compared to common leafy vegetables such as spinach, lettuce, and kale, reflecting strong omega-3 desaturase activity (Barros et al., 2015). Reported concentrations (300–400 mg/100 g FW) exceed those of spinach, previously considered a leading source (Simopoulos and Salem, 1986). While this supports its classification as a functional crop, its nutritional contribution is constrained by low human conversion rates of ALA to EPA (<10%) and DHA (<1%) (Calder 2014). Thus, purslane should be viewed as a complementary, not substitute, source of long-chain omega-3s.

### 6.3 Influence of nitrogen nutrition on lipid metabolism

The relationship between nitrogen fertilization and lipid metabolism in *Portulaca oleracea* L. remains both agronomically important and insufficiently resolved. Mechanistically, nitrogen influences lipid composition through its coupling with carbon metabolism. Empirical evidence suggests a non-linear response. Moderate nitrogen supply generally enhances polyunsaturated fatty acid (PUFA) accumulation, particularly α-linolenic acid (ALA), whereas excessive nitrogen may reduce ALA concentration, indicating an optimum nitrogen level for maximizing fatty acid quality. Fontana et al. (2006) reported increased omega-3 fatty acid accumulation under moderate nitrogen nutrition, whereas Montoya-García et al. (2018) observed declining essential fatty acid concentrations with increasing nitrogen fertilization beyond optimal levels. Likewise, Petropoulos et al. (2019) concluded that both nitrogen source and application rate significantly influence ALA content in purslane, indicating that excessive nitrogen can compromise fatty acid quality. Collectively, these findings indicate that moderate nitrogen availability favors ALA accumulation, whereas excessive nitrogen reduces essential fatty acid concentrations, although the optimum nitrogen level varies with genotype, nitrogen source, harvest stage, and growing environment. Nitrogen form further complicates this relationship. Ammonium-based sources have been linked to higher ALA and lower oxalic acid compared to nitrate-dominant forms, indicating that nitrogen source is a critical determinant of lipid quality (Camalle et al., 2020).

Nitrogen regulation of fatty acid composition is multifaceted, influenced by nitrogen availability, form, and species-specific metabolism. Nitrogen nutrition affects lipid biosynthesis through carbon partitioning and desaturase activity, while nitrogen form can significantly alter the nutritional composition of leafy vegetables, highlighting its importance as a determinant of lipid quality (Rouphael et al., 2018).

### 6.4 Stability of fatty acid profiles across production systems

A key issue in evaluating purslane’s functional value is the stability of its fatty acid profile, particularly ALA, across production, storage, and processing. Its nutritional benefit depends on preserving ALA from field to consumption, yet this is challenged by oxidative degradation driven by light, oxygen, temperature, and lipoxygenase activity factors that disproportionately affect highly unsaturated fatty acids. Evidence indicates that variability in ALA content is shaped more by cultivar, nitrogen management, temperature, and harvest timing than by production system (field vs greenhouse) (Petropoulos et al., 2019; Carrascosa et al., 2023). When these factors are controlled, ALA levels remain comparable across systems, suggesting that management practices, rather than environment type, are the primary determinants (Montoya-García et al., 2018; Petropoulos et al., 2019).

Processing further constrains ALA retention. Thermal methods such as boiling and frying reduce ALA through oxidation, though milder techniques like steaming are less damaging (Srivastava et al., 2019). However, evidence on cooking losses in purslane remains limited despite its widespread culinary use (Cyprian et al., 2017). Drying introduces additional risks, with freeze-drying better preserving lipids and antioxidants than conventional methods (Youssef and Mokhtar, 2014). Overall, purslane’s favorable fatty acid profile is relatively stable but sensitive to controllable factors, including nutrient management, harvest stage, substrate, and processing. Optimizing these variables is essential to maintain its omega-3 value across the supply chain (Carrascosa et al., 2023).

## 7 Bioactive compounds and health-promoting properties

### 7.1 Phenolic compounds, flavonoids, and betalains

Purslane (*Portulaca oleracea* L.) harbors a structurally diverse array of bioactive phytochemicals whose collective pharmacological relevance extends well beyond the species’ established omega-3 fatty acid content. Among these, phenolic compounds, flavonoids, and betalains occupy a central position functioning both as primary antioxidant agents and as modulators of key inflammatory, metabolic, and proliferative signalling pathways.

Total phenolic content (TPC) in purslane extracts, quantified by the Folin-Ciocalteu method and expressed as gallic acid equivalents (GAE), ranges from 0.96 ± 0.04 to 9.12 ± 0.29 mg GAE/g dry weight across reported accessions (Alam et al., 2014a). Structurally, the phenolic fraction of purslane encompasses flavonoids including quercetin, kaempferol, and their glycosidic derivatives alongside phenolic alkaloids termed oleraceins (A, B, C, D, and E), which represent a phytochemically unique class of cyclodopa-derived compounds with demonstrated antioxidant and anti-inflammatory activities (Liu et al., 2021). Oleraceins A and C are present regardless of harvest stage, with reported concentrations ranging from 8.2–103.0 mg/100 g DW and 21.2–143.0 mg/100 g DW respectively (Petropoulos et al., 2019). This sufficiently wide range suggests that cultivation conditions and harvest timing exert substantial influence over their accumulation. Oleracein E has been specifically demonstrated to exert anti-inflammatory effects through reduction of endoplasmic reticulum stress in experimental colitis models (Liu et al., 2021), lending mechanistic specificity to what has otherwise been a broadly characterized anti-inflammatory phenotype in the species. The dopamine and noradrenaline alkaloids present in purslane catecholamines more commonly associated with animal physiology add a further dimension of pharmacological interest, though their functional role in plant physiology and their bioavailability following ingestion remain poorly characterized (Zhou et al., 2015).

Betalains nitrogen-containing pigments comprising betacyanins and betaxanthins constitute a structurally and functionally distinct class of bioactive compounds in purslane that has received comparatively less investigative attention than phenolics and flavonoids, despite possessing potent radical-scavenging activity and demonstrable effects on oxidative stress in animal models (Wang and Yang, 2010; Uddin et al., 2012). The antioxidant contribution of betalains is particularly relevant in the context of purslane’s neuroprotective properties, as betacyanin-rich purslane extracts have been shown to reduce oxidative stress markers and improve cognitive performance in aging mice (Wang and Yang, 2010). However, extrapolation of these findings to human neuroprotection requires considerably more rigorous clinical investigation. Purslane’s total carotenoid content (TCC), ranging from 0.52 ± 0.06 to 5.64 ± 0.09 mg beta-carotene equivalents/g DW (Alam et al., 2014b), further contributes to its antioxidant capacity through singlet oxygen quenching and lipid peroxidation inhibition, with lutein and beta-carotene as the quantitatively dominant species (Li et al., 2024).

### 7.2 Antioxidant, anti-inflammatory, antidiabetic, and cardioprotective activities

The pharmacological activities of purslane are mechanistically anchored in its multi-component antioxidant system encompassing ALA, alpha-tocopherol, ascorbic acid, flavonoids, phenolic alkaloids, betalains, and carotenoids whose synergistic interactions collectively confer a breadth of biological activity that extends across cardiovascular, metabolic, inflammatory, neurological, and oncological pathways (Uddin et al., 2012, 2014; Burri et al., 2017). The antioxidant capacity of purslane has been extensively validated using DPPH radical scavenging, FRAP, and hydrogen peroxide scavenging assays all of which consistently demonstrate high radical-quenching activity attributable to the combined action of its phenolic fraction and lipid-soluble antioxidants (Cai et al., 2003).

Mechanistically, purslane achieves antioxidant protection through multiple complementary pathways: direct radical scavenging by flavonoids and phenolic acids, metal ion chelation that inhibits Fenton-type hydroxyl radical generation, inhibition of lipid peroxidation particularly relevant for protecting the highly oxidation-susceptible ALA fraction and upregulation of endogenous antioxidant enzymes including glutathione peroxidase, catalase, superoxide dismutase, and glutathione reductase (Dkhil et al., 2011; Iranshahy et al., 2017). This multi-target antioxidant profile is more mechanistically comprehensive than that of most conventional vegetables and provides a plausible biochemical foundation for the observed protective effects in disease models. Notably, purslane extracts combined with fish oil have demonstrated enhanced protection against radiation-induced oxidative damage relative to either agent alone (Abd El-Azime et al., 2014). This suggests synergistic interactions between purslane’s plant-derived antioxidants and marine omega-3 fatty acids. Hence this combination has potential clinical relevance for chemotherapy adjuvant protocols that merits further controlled investigation.

The anti-inflammatory activity of purslane is mediated through suppression of multiple proinflammatories signalling cascades. Purslane extracts have been demonstrated to inhibit the NF-κB pathway and reduce the production of TNF-α, IL-6, and IL-1β in inflammatory bowel disease models (Kim et al., 2019; Chen et al., 2022), while simultaneously inhibiting nitric oxide production a key mediator of acute inflammatory responses (Sdouga et al., 2020). In a rat model of LPS-induced lung inflammation, purslane extract at 200–400 mg/kg significantly reduced total and differential white blood cell counts in bronchoalveolar lavage fluid alongside measurable decreases in inflammatory mediator concentrations (Baradaran Rahimi et al., 2019). The purslane-derived alkaloid compound 14-O-acetylcholine demonstrated significant anti-inflammatory activity in a murine model of ulcerative colitis (Wangchuk et al., 2015), while capnoidine prevented chemically induced colitis development in a separate experimental model (Shepherd et al., 2018). These provide compound-specific mechanistic evidence that goes beyond the non-specific anti-inflammatory attribution typical of whole-plant extract studies.

The antidiabetic potential of purslane reflects a multi-pathway mode of action, including improved insulin sensitivity, enzyme inhibition, and oxidative stress reduction. The omega-3 fatty acids in purslane further contribute to antidiabetic outcomes through improvements in lipid metabolism reducing cholesterol, triglycerides, and LDL-cholesterol while increasing HDL-cholesterol (Rideout et al., 2022) through anti-inflammatory suppression of TNF-α and IL-6, cytokines centrally implicated in the development of insulin resistance (Zhou et al., 2015; Rahimi et al., 2018). Although, a meta-analysis of clinical trials has reported that purslane supplementation significantly lowers fasting blood glucose and HbA1c levels (Iranshahi et al., 2017). These findings are weakened by small sample sizes, heterogeneity in dosage, and short intervention periods, limiting generalizability Abbasi et al. (2023).

The cardioprotective potential of purslane is primarily attributable to its ALA content, which has been demonstrated to reduce plasma triglycerides and LDL-cholesterol while increasing HDL-cholesterol in experimental models. This is a lipid-modulating effect consistent with the established cardiovascular benefits of dietary omega-3 fatty acids (Sala-Vila et al., 2022). A clinical trial in patients with non-alcoholic fatty liver disease a condition closely linked to cardiometabolic risk demonstrated that purslane supplementation improved liver function parameters, metabolic profile, and oxidative stress biomarkers (Milkarizi et al., 2024), providing an example of human clinical evidence supporting purslane’s systemic cardiometabolic benefits.

### 7.3 Evidence from *In Vitro*, animal, and limited human studies

The evidence base for purslane’s health effects is heavily skewed toward *in vitro* and animal studies, creating a clear gap between mechanistic plausibility and clinical certainty. While bioactive compounds show strong antioxidant, anti-inflammatory, and metabolic effects experimentally, human evidence remains limited, heterogeneous, and focused on surrogate outcomes rather than definitive clinical endpoints.

*In vitro* studies have been valuable for identifying mechanisms such as enzyme inhibition, cytotoxicity, and radical scavenging but their predictive relevance is constrained by non-physiological conditions, including high compound concentrations and lack of a metabolic context (Cai et al., 2003; Patil et al., 2012). The frequent omission of bioavailability and biotransformation considerations further weakens their translational value (Uddin et al., 2014). Animal studies provide more integrated evidence, demonstrating effects such as neuroprotection, hepatoprotection, and anti-inflammatory activity across various disease models (Peng et al., 2019). However, these findings are limited by species differences and the use of high extract doses that are not achievable through normal dietary intake. As a result, extrapolation to human health benefits is often overstated.

## 8 Functional food applications and food system integration

### 8.1 Criteria for functional food classification

The classification of purslane (*Portulaca oleracea* L.) as a functional food rest on its capacity to deliver measurable health benefits beyond basic nutritional sustenance, a criterion if, applied rigorously, demands more than compositional richness alone. Functional food designation requires demonstrated bioactivity of specific compounds at physiologically achievable dietary intakes, evidence of health benefit in target populations, and acceptable safety profiles across realistic consumption patterns (Nemzer et al., 2020; Kumar et al., 2021). Against these criteria, purslane presents a compelling case. Its multi-component bioactive profile encompassing ALA, alpha-tocopherol, ascorbic acid, carotenoids, flavonoids, oleraceins, and polyphenols satisfies the compositional dimension of functional food classification (Uddin et al., 2014; Petropoulos et al., 2019). However, as critically discussed in Section 7, the translation of this compositional richness into demonstrated health outcomes at dietary doses in human populations requires further investigations.

A functional food ingredient must deliver a consistent and quantifiable dose of its active compound. The near-tenfold variation in total phenolic content (0.96–9.12 mg GAE/g DW) and the harvest-stage dependence of ALA concentrations documented across purslane studies (Alam et al., 2014a; Petropoulos et al., 2019) indicate that the crop, as currently cultivated and marketed, might meet this standardization criterion. However, targeted agronomic and post-harvest management protocols must be specifically designed to maintain compositional consistency.

### 8.2 Culinary uses and value-added product development

Purslane has a well-documented history of culinary incorporation across Mediterranean, Middle Eastern, Asian, and African food traditions, where it is consumed as a raw salad ingredient, cooked green our used as a soup constituent. These applications exploit its mucilaginous texture, mild tangy flavor, and succulent palatability (Carrascosa et al., 2023). Its integration into tomato-based sauces has been demonstrated to enhance protein content while modifying total soluble solid profiles (Apostol et al. 2020). This illustrates its functional value-adding potential as a fortifying ingredient in processed food matrices. Beyond fresh consumption, emerging research has explored purslane’s incorporation into bread, yogurt, and nutraceutical extract formulation product categories that benefit from the plant’s antioxidant, antimicrobial, and lipid-enriching properties (Makangali et al., 2024).

However, a critical limitation of value-added product development involving purslane is the thermal sensitivity of its most nutritionally distinctive components particularly ALA and ascorbic acid to the processing temperatures typical of baking, pasteurization, and extrusion (Youssef and Mokhtar, 2014). Freeze-drying has been identified as the processing technology that causes least damage to purslane’s antioxidant and lipid profile (Youssef and Mokhtar, 2014), but its high capital and operational cost limits scalability for commodity food applications. The development of processing protocols that balance ALA preservation, oxalic acid reduction, microbiological safety, and sensory acceptability simultaneously represent the primary technical challenge in advancing purslane-based value-added product development. It is a challenge that has not yet been systematically addressed in the literature. Product developers must therefore navigate a trade-off landscape in which the interventions most effective at neutralizing anti-nutritional factors (heat treatment) are simultaneously the most damaging to the omega-3 fatty acid and vitamin fractions that constitute purslane’s principal functional food value proposition.

### 8.3 Nutritional labelling and consumer relevance

The consumer relevance of purslane as a functional food is contingent not only on its bioactive composition but on the effective communication of its nutritional attributes through labelling and public health messaging dimensions that are substantially underdeveloped relative to the scientific evidence base. However, this nutritional argument is undermined by the low and variable conversion efficiency of dietary ALA to EPA and DHA in humans (Calder, 2014). This a metabolic reality that complicates the use of its ALA content as the primary basis for cardiovascular or cognitive health claims on functional food labels.

Effective nutritional labelling for purslane-based products must therefore communicate the distinction between ALA and long-chain omega-3 fatty acids with scientific accuracy, position purslane’s omega-3 contribution within the context of overall dietary fat quality and clearly indicate the serving-size-dependent nature of micronutrient and phytochemical contributions. The challenge is compounded by the compositional variability documented across studies as a 100 g serving of purslane may deliver ALA concentrations ranging from 300 mg to over 500 mg depending on cultivar, production conditions, and harvest timing (Barros et al., 2015). This makes generic label claims inaccurate without batch-specific compositional verification. Consumer acceptance of purslane as a functional food ingredient is further constrained by its residual perception as a weed in many temperate food cultures (Uddin et al., 2014) and its historical association with subsistence-level or low-income diets in others. Hence the development of effective consumer communication strategies including culinary education, recipe development, and retail positioning alongside established functional leafy greens represents a market development priority that science alone cannot address.

### 8.4 Anti-nutritional factors and mitigation strategies

The functional food potential of purslane is materially qualified by the presence of two principal anti-nutritional factors oxalic acid and nitrates whose accumulation in leaf tissue under certain production and nutritional management conditions poses genuine dietary safety concerns that must be critically evaluated rather than minimized in the functional food discourse (Carrascosa et al., 2023).

Oxalic acid, present in purslane leaves at 671–869 mg per 100 g FW (Uddin et al., 2014) and reaching as high as 8.6 g per 100 g FW at later harvest stages (Petropoulos et al., 2019), exerts its anti-nutritional effect primarily through the formation of insoluble calcium oxalate complexes that reduce the bioavailability of dietary calcium and, at very high intakes, may contribute to urinary oxalate load and kidney stone risk in susceptible individuals (Srivastava et al., 2019). Early harvesting (approximately 29–35 DAS), which is associated with higher α-linolenic acid (ALA) concentrations, also results in lower oxalic acid levels than later harvest stages (Petropoulos et al., 2019). This favorable combination enhances the nutritional quality of purslane and can be incorporated into agronomic management recommendations. Purslane contains both soluble (free or potassium/sodium-bound) and insoluble (calcium-bound) oxalates. The insoluble calcium-bound fraction is particularly important for calcium bioaccessibility because it binds calcium into poorly absorbable calcium oxalate complexes, whereas the soluble fraction is more closely associated with intestinal oxalate absorption and kidney stone risk (de Souza et al., 2024). Thermal processing, particularly boiling, preferentially removes soluble oxalates through leaching into the cooking water, whereas the insoluble fraction is comparatively resistant and only partially reduced (Srivastava et al., 2019). Consequently, combining early harvesting with appropriate cooking practices provides a practical strategy to reduce the overall oxalate burden while improving calcium bioaccessibility. However, thermal processing may also reduce ALA and heat-sensitive vitamins, highlighting the need to optimize cooking conditions to maximize nutritional quality.

Nitrate accumulation in purslane leaf tissue represents a distinct but equally significant food safety concern, particularly given the species’ capacity to accumulate nitrate under high nitrogen fertilization regimes a tendency shared with other succulent leafy vegetables. Kaymak (2013) demonstrated a direct proportional relationship between nitrogen fertilizer application rate and tissue nitrate concentration in purslane, with excessive nitrogen fertilization producing nitrate levels that may approach or exceed regulatory thresholds for leafy vegetables in some jurisdictions. Human metabolism converts dietary nitrate to nitrite through oral bacterial reduction, and while low-level nitrate consumption from vegetables is not considered harmful and may indeed confer cardiovascular benefits through nitric oxide generation, excessive dietary nitrate intake has been associated with methemoglobinemia risk in infants and potential carcinogenic nitrosamine formation under certain conditions (Santos et al., 2016; Fallah and Omrani, 2019). The application of ammonium sulfate as a nitrogen source has been identified as the fertilizer formulation producing the lowest nitrate accumulation in purslane among tested nitrogen sources (Kaymak, 2013), providing a practically actionable mitigation strategy. More broadly, integrated nitrogen management incorporating split application, timing aligned with plant uptake capacity, organic nitrogen supplementation, and harvest scheduling that avoids peak nitrate accumulation periods constitutes the most effective systemic approach to managing nitrate risk without sacrificing yield or nutritional quality (Fallah and Omrani, 2019).

## 9 Sustainability, climate resilience, and food security implications

The sustainability credentials of purslane (*Portulaca oleracea* L.) as a food crop derives principally from its exceptional tolerance of resource-limited growing conditions. This characteristic positions it favorably against conventional leafy vegetables in the context of mounting pressures on agricultural inputs, water availability, and arable land productivity (Carrascosa et al., 2023). Purslane’s C_4_/CAM-intermediate photosynthetic physiology confers it with inherently superior water-use efficiency relative to C_3_ leafy crops under high-temperature and high-irradiance conditions (Kopsell et al., 2016). Its succulent tissue architecture on the other hand enables it to store substantial amounts of water that buffers the plant against short-term soil moisture deficits without yield penalties of the magnitude observed in non-succulent species (Uddin et al., 2014). These physiological attributes translate into a demonstrably lower irrigation requirement per unit of edible biomass produced. This is a significant advantage in water-scarce production environments and an underexploited argument for purslane’s inclusion in low-input and climate-adapted cropping systems.

Purslane’s prolific seed production with individual plants generating up to 10,000 seeds under favorable conditions (Jin et al., 2016) and its capacity for vegetative propagation from stem cuttings further reduces production input requirements by minimizing seed purchase costs and enabling low-cost crop multiplication. These reproductive characteristics, combined with its rapid establishment and short production cycle of 35–65 days from sowing to harvest (Alam et al., 2014a; Karkanis and Petropoulos, 2017), support a high frequency of cropping cycles per unit area per year. This provides a yield intensity advantage that partially offsets purslane’s lower per-plant biomass generation relative to larger leafy vegetable species.

Perhaps the most strategically significant dimension of purslane’s sustainability profile is its demonstrated capacity to produce nutritionally valuable biomass in soil and climatic conditions that preclude economically viable production of most conventional vegetables. Purslane exhibits substantial tolerance of soil salinity which is a stress factor that increasingly constrains agricultural productivity globally due to soil degradation, salt accumulation in soils from irrigations water, and saltwater intrusions into coastal agricultural lands (Uddin et al., 2012; Jin et al., 2016). While salinity reduces overall growth and biomass yield in purslane, it concurrently enhances ALA accumulation which is an adaptive response to membrane lipid unsaturation under osmotic stress (He et al., 2021). This creates a paradoxical situation in which agronomically marginal saline conditions may yield purslane with superior omega-3 nutritional quality relative to optimal-condition production.

The capacity of purslane to grow productively in poor, compacted, and drought-prone soils enabled by its taproot system with fibrous secondary roots and its CAM-like carbon assimilation under water stress extends its productive range to dryland and semi-arid environments where leafy vegetable production is conventionally unfeasible (Chugh et al., 2019). The food security significance of purslane’s omega-3 contribution can be pronounced for both developing and developed nations. However, due to the high cost of purchasing marine-derived products rich in omega-3 fatty acids in developing countries, purslane can serve as a cheaper alternative (Uddin et al., 2014; Popescu et al., 2018). While the metabolic conversion efficiency of ALA to EPA and DHA is low estimated at less than 10% and 1% respectively under typical human physiological conditions (Calder 2014) regular consumption of ALA-rich foods remains an established strategy for maintaining omega-3 status in plant-based diet adherents. As such, the addition of purslane to existing plant-based food systems could meaningfully augment ALA intakes without requiring dietary pattern restructuring beyond the substitution for less nutritionally dense leafy greens.

## 10 Research gaps and future directions

Despite expanding evidence on purslane’s nutritional properties, the molecular mechanisms through which nitrogen regulates fatty acid biosynthetic pathways remain critically under-characterized. While directional correlations between nitrogen rate and individual fatty acids including positive associations with ALA and negative associations with linoleic and stearic acids have been documented (Petropoulos et al., 2019), the enzymatic control points governing omega-3 desaturation, particularly chloroplastic FAD7, FAD8, and endoplasmic reticulum FAD3 desaturases, have not been characterized under varying nitrogen regimes in purslane. Future research must prioritize transcriptomic profiling of nitrogen-responsive lipid metabolism genes and metabolomic integration of nitrogen assimilation and lipid biosynthesis pathways to transform current descriptive observations into mechanistically grounded agronomic interventions (Wasternack and Feussner, 2018).

The cultivar-dependent variability in purslane’s nutritional response to nitrogen fertilization spanning wild ecotypes to ornamental morphotypes such as ‘Goldberg Golden’ represents a major gap that fundamentally limits the transferability of existing agronomic recommendations (Alam et al., 2014a). Systematic multi-cultivar, multi-environment factorial trials incorporating diverse ecotypes across nitrogen rate gradients and measuring comprehensive nutritional panels simultaneously are needed.

The integration of plant breeding and nutrient management as complementary strategies for nutritional quality enhancement remains largely unexplored. Current breeding efforts have focused primarily on agronomic traits, while the genetic architecture of ALA biosynthesis, mineral accumulation efficiency, and nitrogen use efficiency in purslane remains essentially uncharacterized at the genomic level (Karkanis and Petropoulos, 2017). Development of genomic resources including reference genome assemblies and QTL maps for key nutritional traits would enable marker-assisted selection for high-ALA, low-oxalate, nitrogen-efficient ideotypes whose performance is robust across diverse production environments, creating the genotype-by-nutrition interaction framework currently absent from purslane research.

Post-harvest quality management and human bioavailability represent the most consequential and least investigated dimensions of purslane’s functional food value chain. The fate of key bioactive compounds ALA, phenolics, oleraceins, minerals, and vitamins through storage, domestic preparation, gastrointestinal digestion, and systemic absorption in humans remains largely unquantified (Youssef and Mokhtar, 2014). Critical unresolved questions include the bioavailability of calcium and iron in the presence of endogenous oxalates, the minimum effective dietary dose required to produce measurable changes in plasma omega-3 indices, and the stability of oleracein alkaloids through cooking and digestion (Uddin et al., 2014; Calder 2014; Srivastava et al., 2019). Addressing these gaps requires coordinated research spanning *in vitro* digestion modelling, human pharmacokinetic studies, and adequately powered randomized controlled trials with validated biomarker endpoints.

## 11 Conclusion

This review identifies purslane (*Portulaca oleracea*, L*.)* as a strategic crop for advancing nutrition and resilient food systems, given its high content of Omega-3 fatty acids (ALA), vitamins, minerals, and bioactive compounds. Its adaptability to marginal environments further supports its role in food security. However, nutritional quality remains highly variable, governed by genotype, environmental conditions, and nutrient management, with nitrogen exerting inconsistent effects on nutrient density and phytochemical accumulation. Persistent limitations including non-standardized methodologies and insufficient resolution of genotype × environment interactions restrict cross-study comparability and agronomic precision. Future research should prioritize integrated, multi-factorial designs, standardized analytical frameworks, nutrient bioavailability, and post-harvest optimization, alongside economic and consumer validation. Advancing such coordinated approaches is essential to fully position purslane as a scalable, nutrient-dense solution to global dietary challenges.
